# Gastrostomy for infants with severe epidermolysis bullosa simplex in neonatal intensive care

**DOI:** 10.1186/s13023-021-01896-0

**Published:** 2021-06-11

**Authors:** M. Marro, S. De Smet, D. Caldari, C. Lambe, S. Leclerc-Mercier, C. Chiaverini

**Affiliations:** 1 Department of Dermatology, CRMRPM-Sud, Université Côte d’Azur, Centre Hospitalier Universitaire de Nice, 06200 Nice, France; 2 Department of Neonatal Intensive Care, Université Côte d’Azur, Centre Hospitalier Universitaire de Nice, Nice, France; 3 Department of Pediatric Gastroenterology, Centre Hospitalier Universitaire de Nantes, Nantes, France; 4 Department of Pediatric Gastroenterology, Hôpital Universitaire Necker– Enfants Malades, Assistance Publique – Hôpitaux de Paris-Centre (AP-HP), Paris, France; 5 Department of Dermatology, Reference Center for Genodermatoses and Rare Skin Diseases (MAGEC), Hôpital Universitaire Necker– Enfants Malades, Assistance Publique – Hôpitaux de Paris-Centre (AP-HP), Paris, France

**Keywords:** Epidermolysis bullosa, Infant, Gastrostomy

## Abstract

**Introduction:**

Severe epidermolysis bullosa simplex (EBS sev) is a rare genodermatosis characterized by congenital generalized blistering and mucosal involvement. Increased needs and decreased intake quickly lead to nutritional imbalance. Enteral nutrition support is proposed, but classical nasogastric tubes are not well tolerated in these patients and gastrostomy is preferred.

**Objective and methods:**

To report the experience with EBS sev in neonatal units of French reference centers for gastrostomy. In this retrospective multicentric study, we included all patients with EBS sev who had gastrostomy placement before age 9 months during neonatal care hospitalization.

**Results:**

Nine infants (5 males/4 females) with severe skin and mucosal involvement were included. A gastrostomy was decided, at an early age (mean 3.7 months, range 1.4 to 8 months) in infants with mean weight 4426 g (range 3500 to 6000 g). Techniques used were endoscopy with the pull technique for 5 infants and surgery under general anesthesia for 4. Main complications were local but resolved after treatment. All infants gained weight after gastrostomy. The mean withdrawal time (n = 7) for the gastrostomy was 35.8 months (range 10.5 months to 6.5 years). Seven children had persistent oral disorders.

**Conclusions:**

Gastrostomy in infants with EBS sev can be necessary in neonatal intensive care units. Both surgical and endoscopic pull techniques seem efficient, with good tolerance.

Dear editor,

Severe epidermolysis bullosa (EB) simplex (EBS sev), the most severe form of EB simplex, is characterized by generalized blistering and mucosal involvement [1]. In the neonatal period, patients have an increased protein loss due to cutaneous involvement and feeding difficulties secondary to the mucosal involvement and sedation induced by analgesics, which leads to nutritional imbalance [2]. Enteral nutrition, most often with a nasogastric feeding tube is started but with poor tolerance. Gastrostomy is then proposed, but medical data are lacking in literature.

In this retrospective multicentric French study, we included 9 infants (5 males) with EBS sev, who had gastrostomy placement before age 9 months (Table 1). All infants had severe skin (> 25% of surface area) and mucosal involvement leading to their admission in a neonatal intensive care unit (Fig. 1). Analgesic treatments included paracetamol (n = 9), morphine (n = 9) ketamine (n = 6) and amitriptyline (n = 3). All infants had feeding difficulties. Clinical gastroesophageal reflux was observed in 8/9 infants and treated with esomeprazole. Enteral feeding nutrition with a nasogastric feeding tube, to reach an objective of caloric intake of 130 kcal/kg/day, was not well tolerated because of the inability to correctly attach the tube to the skin, which led to its frequent pulling out and the mucosal fragility leading to blisters secondary to the rubbing of the tube. A gastrostomy was then decided, at an early age (mean 3.7 months) in infants with mean weight 4426 g. Techniques used were endoscopy (n = 5) or surgery (laparoscopy) (n = 4) without immediate complication. Wound healing difficulties around the gastrostomy hole (n = 2), pyogenic granuloma (n = 3) and vomiting (n = 5) were reported. Appropriate treatment enabled the rapid resolution of these complications. All infants gained weight after the gastrostomy up to the third centile for 7 infants. They continued to have oral alimentation, with persistent oral disorders for 7. Gastrostomy was removed after a mean duration of 35.8 months in 8 children. One child still had their gastrostomy at 11 years.

**Table 1:** Characteristics of patients with gastrostomy

	Patient 1	Patient 2	Patient 3	Patient 4	Patient 5	Patient 6	Patient 7	Patient 8	Patient 9
Sex	Male	Female	Female	Male	Male	Female	Male	Male	Female
Genetic mutation	KRT14	KRT5	KRT14 + KRT5	KRT5	KRT5	KRT5	ND	KRT5	KRT 14
Birth weight (g)	3190 (53rd centile)	2940 (25th centile)	2830 (5th centile)	3040 (60th centile)	3000 (19th centile)	2360 (53rd centile)	3600 (50th centile)	2590 (2nd centile)	4140 g (99th centile)
Hospitalisation length (months)	2	5	7	4.5	5	4	6	4.5	7
Gastrostomy technique	Endoscopy (pull)	Endoscopy (pull)	Endoscopy (pull)	Surgery	Endoscopy (pull)	Surgery	Surgery	Surgery	Endoscopic (pull)
Age at gastrostomy	1 month 13 days	3 months 25 days	8 months	2 months	2 months 11 days	5 months	4 months	3 months	4 months
Weight at gastrostomy (g)	3500	4500	6000	4850	4000	4250	5010	3300	4030 g
Age at gastrostomy withdrawal	12 months	15 months 7 days	6 years 3 months	36 months	20 months	No withdrawal	39 months	36 months	6 years 9 months
Complications	GERD granuloma tissue vomiting oral disorders	GERD vomiting	GERD oral disorders	GERD vomiting granuloma tissue	Oral disorders	Oral disorders	GERD vomiting oral disorders	GERD vomiting granuloma tissue leaking oral disorders	GERD

Summary of demographic, genetic and gastrostomy information

*GERD* gastroesophageal reflux disease, *ND* not determined

**Fig. 1:**
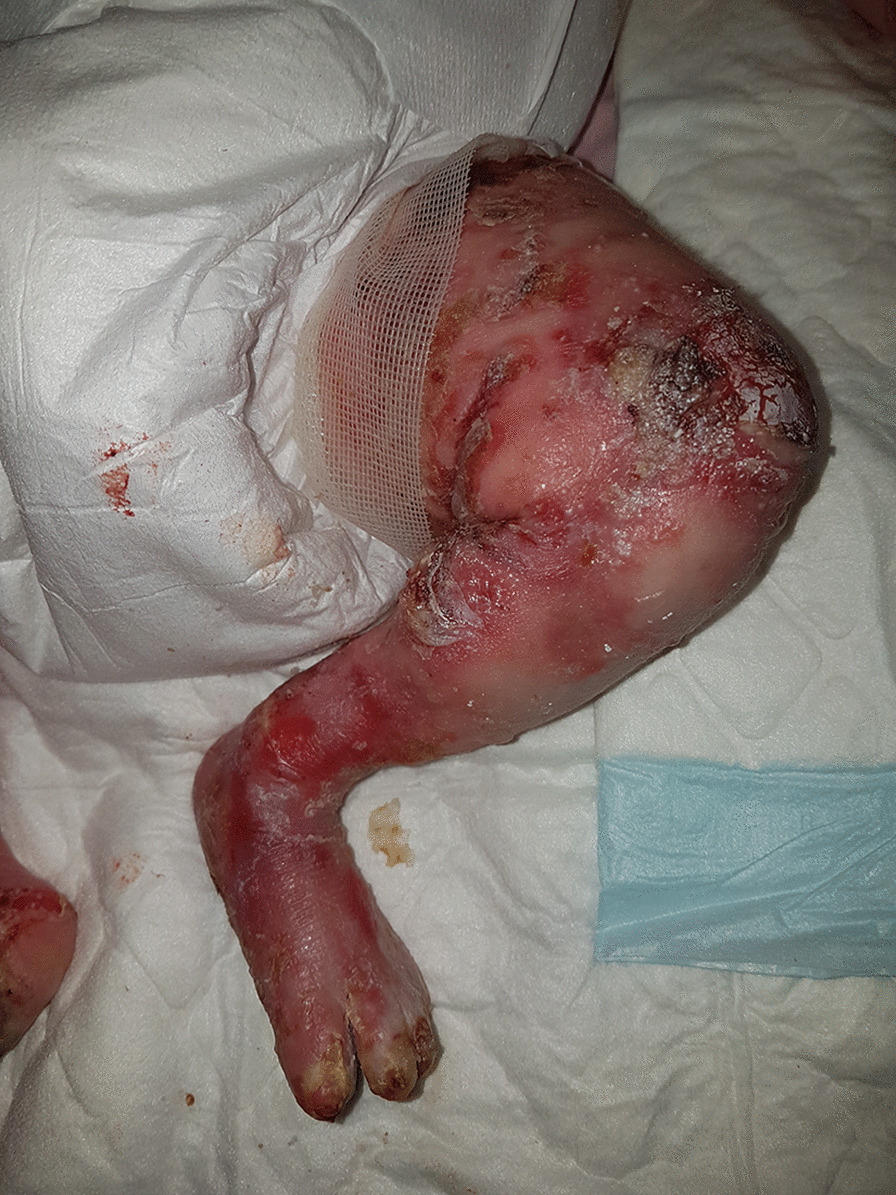
Severe cutaneous involvement in a 2 month old infant with EBS severe

Gastrostomy for children is indicated in case of long-term inadequate intake [3] and can be placed with different techniques: percutaneous under endoscopy (PEG), surgery or percutaneous under radioscopy (PER) [4]. This last technique is not used in newborns. In EB patients, gastrostomy tube placement is required mostly for severe junctional or dystrophic subtypes and usually in childhood or adulthood [5] due to the progressive worsening of their conditions. For these patients with severe mucosal involvement and risk of oesophageal strictures, the PEG technique is not indicated and the PER method is usually preferred, but the laparoscopic approach has been used successfully [6]. In contrary, patients with EBS sev, can have severe phenotype during infancy with progressive improvement with time. Furthermore, mucosa involvement usually spare their oesophagus [2]. Then, if indications for gastrostomy are the same, the paradigm is different. The young age of the patients contraindicates the PER technique, but the absence of esophageal involvement allows for the PEG technique. Of note, 4 of our 9 infants underwent surgical insertion of gastrostomy without severe complications. This technique seems to be useful when PEG is not available. Concordant with the literature, complications occurred in 55% of our infants, with vomiting and local anomalies, with no difference between the PEG and surgical method [7]. As for other EB subtypes, we found a positive nutritional impact of gastrostomy placement on weight gain and no difference between methods of insertion [8]. According to the natural improvement of the disease, in 7/9 infants, the gastrostomy tube could be withdrawn, before age 3 years in 6 cases. Seven children had persistent oral disorders.

In conclusion, gastrostomy can be necessary for infants with EBS sev. Both surgical and endoscopic pull techniques seem efficient, with good tolerance.

## Data Availability

The datasets used and/or analyzed during the current study are available from the corresponding author on reasonable request.

## Abbreviations

EBS sev: Severe pidermolysis bullosa simplex
PEG: Percutaneous under endoscopy
PER: Percutaneous under radioscopy
